# Conversation Analysis Based Simulation (CABS): A method for improving communication skills training for healthcare practitioners

**DOI:** 10.1111/hex.13834

**Published:** 2023-08-17

**Authors:** Alison Pilnick, Rebecca O'Brien, Suzanne Beeke, Sarah Goldberg, Megan Murray, Rowan H. Harwood

**Affiliations:** ^1^ School of Sociology and Social Policy, University of Nottingham Nottingham UK; ^2^ School of Health Sciences, University of Nottingham UK and Nottinghamshire Healthcare NHS Foundation Trust Nottinghamshire UK; ^3^ Division of Psychology and Language Sciences University College London London UK; ^4^ School of Health Sciences University of Nottingham Nottingham UK; ^5^ Simulated Patients Workshop Team Nottingham UK; ^6^ School of Health Sciences, University of Nottingham UK and Nottingham University Hospitals NHS Trust Nottingham UK

**Keywords:** authenticity, communication skills training, conversation analysis, healthcare practitioners, simulated patients, simulation

## Abstract

**Background:**

Actors portraying simulated patients are widely used in communication skills training in healthcare, but debates persist over the authenticity of these interactions. However, healthcare professionals value simulation‐based training because of the opportunity to think and react in real time, which alternatives cannot provide.

**Objective:**

To describe a method for the use of simulation which maximises authenticity by grounding training in real, observed, patterns of patient communication.

**Design:**

Naturally occurring care interactions were video recorded and analysed using conversation analysis (CA) to identify communication patterns. We focused on sites of recurring interactional trouble as areas for training, and identified more and less effective ways of dealing with these. We used the CA findings to train actors portraying simulated patients, based on the observed interactional patterns.

**Settings and Participants:**

Patients living with dementia and healthcare practitioners (HCPs) on two acute healthcare of the elderly wards in the English East Midlands.

**Outcome Measures:**

One month later HCPs reported using the skills learned in clinical practice. Masked‐ratings of before and after simulated patient encounters confirmed these self‐reports in relation to one key area of training.

**Results:**

The Conversation Analysis Based Simulation (CABS) method used in this setting showed positive results across a range of quantitative and qualitative outcome measures. What is significant for the transferability of the method is that qualitative feedback from trainees highlighted the ability of the method to not only illuminate their existing effective practices, but to understand why these were effective and be able to articulate them to others.

**Discussion/Conclusion:**

While the CABS method was piloted in the dementia care setting described here, it has potential applicability across healthcare settings where simulated consultations are used in communication skills training. Grounding simulated interaction in the observed communication patterns of real patients is an important means of maximising authenticity.

**Patient and Public Contribution:**

The VideOing to Improve dementia Communication Education (VOICE) intervention which piloted the CABS method was developed by a multidisciplinary team, including three carers of people with dementia. People living with dementia were involved in the rating of the before and after video simulation assessments.

## 1 INTRODUCTION

Whilst consultations with simulated patients are widely used in the training and assessment of healthcare practitioners (HCPs), there is ongoing debate over how far these consultations reflect real clinical encounters.^1^, ^2^, ^3^ The idea of authenticity, or the lack of it, is key to critiques of the approach^4^; it has been suggested that inauthentic simulations can lead to embarrassment, or to trainees behaving differently in ways that can impact the learning process.^1^ This has resulted both in recommendations as to how sociolinguistic methods can be used to better evaluate communication skills training (CST) in healthcare^5^ and also the proposal of alternative methods for training which avoid the use of professional actors trained as simulation practitioners.^1^, ^6^ However, as we have previously argued based on a scoping review of the use of simulated interaction in CST,^7^ consultations with simulated patients contain a number of aspects which healthcare practitioners identify as valuable skills development opportunities. The experiential learning interactions that are made possible through simulation include the ability to think and respond in real time as part of an actual interaction, the ability to receive immediate feedback on the simulated consultation, and the ability to watch and learn from the way fellow professionals approach the same simulation task in real time.^7^ In this paper we demonstrate that, whilst sociolinguistic methods can undoubtedly be useful in the evaluation of simulation, they can also be used in its development and delivery. Using the example of a CST intervention developed as part of a UK National Institute for Health Research‐funded study to improve communication in dementia care on acute hospital wards,^8^, ^9^ we show how the sociological method of conversation analysis (CA) can be used successfully in the training of simulation practitioners. We call this approach CABS: Conversation Analysis Based Simulation. Whilst the setting for our development of CABS was acute care in a hospital environment, the principles of this intervention are transferable to any healthcare setting in which simulation is used as part of CST.

The term ‘simulation’ can be used in more than one sense in the literature, sometimes conflated with role play, and sometimes used to describe various sorts of virtual reality environments which have been created to allow HCPs to practice aspects of their roles. In this paper, and in the name of our approach, we use the term simulation to describe CST sessions where professional actors who have been trained in simulation techniques take on the patient role. This is distinct from role play where fellow learners take the part of a patient. As Wong et al.^10^ ^,p.513^ describes, the aim is to ‘provide realistic presentations of patients with specified conditions to allow students to experience the real sense of treating a patient’.

CA is a well‐established method for analysing communication and social interaction. It is a sociological method for the detailed study of interaction, which also draws on insights from linguistics and psychology.^11^ It has been widely used in healthcare in the development of a range of successful CST interventions, in fields such as stroke,^12^, ^13^ psychosis,^14^, ^15^ primary care^16^ and end‐ of‐ life care.^17^ For example, Beeke and colleagues developed the Better Conversations with Aphasia approach to training communication partners and people with stroke‐related communication difficulties to communicate better with each other.^13^ The approach was based on empirical work using CA to compare the communication of video‐recorded family members before and after training. Beeke et al. were able to characterise strategies that were beneficial for communicating with people with aphasia, and evaluation of the training emerging from this revealed a significant change in strategy use, and in positive evaluations of interaction.^18^

Conversation Analysis has also been used to improve CST by incorporating videos and transcripts of real interaction as part of the training sessions. Stokoe^1^, ^6^ developed the Conversation Analytic Role Play Method (CARM) specifically as a response to perceived inauthenticity in role play and simulated interaction. The CARM method involves trainees watching videos of interaction but stopping the video at certain points to discuss it as it unfolds, considering for example what their possible responses might be to a particular utterance or scenario before going on to see how this was managed by the practitioner in the recording. Role play between participants is used to practice alternative responses. This approach has been used in settings such as neurology, helping neurologists to distinguish between epileptic and non‐epileptic seizures by identifying linguistic features in the way patients described their symptoms.^19^

Whilst the CARM method has undoubtedly been beneficial in some settings, it could however be argued that it retains a degree of the inauthenticity that it was developed to counter. This is because trainees have time to consider and discuss their responses, which is a feature that is not available in real‐time patient encounters. It has been suggested by other researchers that CA could instead be used to identify patterns in communication to create more authentic role play and simulated interaction scenarios.^7^, ^20^ In this paper, we outline our development and use of the CABS approach which we have created to do this.

## 2 MATERIALS AND METHODS

The materials we used to develop our approach were collected as part of a study funded by NIHR Health Services and Delivery Research (ref 13/114/93), entitled ‘VideOing to Improve dementia Communication Education’ (VOICE).^9^ The objective of the study was to design and evaluate a communication training intervention for healthcare professionals caring for people with dementia in acute hospitals. We received ethical approval from the Yorkshire and Humber—Bradford Leeds Research Ethics Committee (ref 15/YH/0184). The training developed for HCPs, the ‘VOICE for dementia’ training programme, was underpinned by the CABS approach, and was subsequently delivered beyond the initial (regional) training sites with funding from Health Education England. Our multidisciplinary team included three carers for people living with dementia, who were involved throughout the intervention development, as well as doctors, nurses, and speech and language therapists (SLTs). The course was piloted on six experienced healthcare practitioners who gave advice on how it could be refined to be more acceptable and relevant to the needs of HCPs. Other members of a wider Public and Patient Involvement (PPI) group with experience of dementia were involved in the end of course evaluation process described below.

HCPs were recruited from Healthcare of the Older Person wards at a large teaching hospital in the East Midlands of England. We aimed to video record 40 care‐giving encounters, which was estimated would give around 6 h of recorded interaction. We recruited HCPs willing to be video recorded in advance of recruiting patient participants, though HCPs were only ultimately video recorded if we were able to recruit a patient participant in their care. Forty‐one HCPs were recruited; these included doctors (*n* = 12), nurses (including mental health nurses) (*n* = 19) and allied health professionals (physiotherapists, SLTs, and occupational therapists) (*n* = 10). Of these, 26 were ultimately video recorded for the study. Twenty‐seven patients were recruited to the study, and of these, 26 were filmed. None of the patients recruited had the capacity to give informed consent. We followed the requirements of the Mental Capacity Act (2005) and sought consultee advice from a family member or friend to include them in the study. Patients could be filmed more than once, with a different HCP, so some appeared on up to three occasions. In total 41 encounters were recorded, with an average recording length of 9.24 min. For a CA analysis, length or duration of interaction is less important than having a data set which will allow comparative analysis, for example almost all of our recordings contained repeated instances of practitioners making requests.

The range of HCPs that we recruited meant our data set included a wide range of caregiving activities. These included giving medication, supporting mealtimes, changing wound dressings, and conducting a variety of clinical assessments. For ethical reasons, we did not record interactions involving intimate personal care. For the first phase of the study, we conducted a CA analysis of these recorded interactions. We carried out our analysis according to the established principles of the method,^21^ including the use of detailed transcription according to Jeffersonian conventions^22^ and regular data sessions both between the research team and with other experienced CA researchers to develop and test findings. Given the practice‐improvement focus of the project, our initial aim was to identify instances and contexts where there were recurring interactional difficulties. With the constraints of time and funding, our analysis was also guided by the principle of utility for HCPs, that is, it was centred around issues that were likely to be problematic in practice and therefore had the potential to provide relevant ‘trainables’. We use the term ‘trainable’ here, as we have used it throughout our training materials, to mean an aspect of communication practice that HCPs can be trained in. This analysis was also informed by a systematic review of dementia communication training skills carried out at the beginning of the project,^23^ and led us to focus initially on two specific areas: requests and refusals^24^ and negotiating the closing of care encounters.^25^ Further work when the initial project had finished focused on how HCPs respond to hard‐to‐interpret talk from people living with dementia,^26^ which has subsequently been incorporated into the training. In examining all of these areas, and in line with the principles of a CA approach, we began by identifying and categorising patterns of interaction, grouping observations about practices in the data, and establishing the specific communicative practices involved. An example of a CA transcript used in our analysis is given in Table 1.

**Table 1:** Example of a CA transcript from the VOICE for dementia study.

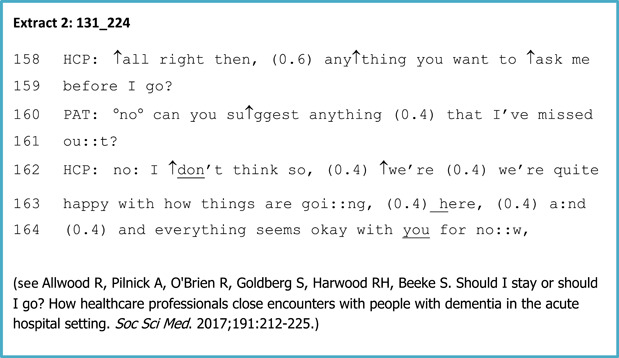		

We then considered which of these practices were associated with interactional troubles or challenges, and which appeared to be effective in achieving particular goals. Effectiveness in this context was judged interactionally, for example improving patient understanding, gaining co‐operation, or easing distress or anxiety. This is the CABS process by which our trainables were identified and agreed upon.

## 3 RESULTS

**Figure 1:**
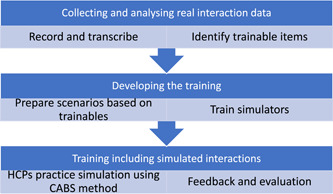
Overview of the steps in the Conversation Analysis Based Simulation (CABS) process. HCP, healthcare practitioner.

Figure 1 gives an overview of the process of transforming the trainables into a simulation‐based CST intervention using CABS. The training course and materials were developed and refined over four whole‐day intervention development meetings. Present at these meetings were the researchers, family carers of people living with dementia, experts at working with simulated patients in education, an actor with significant experience of patient simulation in healthcare, educational experts, and HCPs with expertise in caring for patients with dementia.

### 3.1 Selecting video extracts for use in training

Following the identification of our trainables, the next stage in our process was to select appropriate extracts from the videos for use in training. For example, from our data set, we collected scenarios where requests were made in different ways by HCPs, and how they were responded to. Examples of these included imperatives (e.g. ‘I need you to do X’), collaboratively framed requests (e.g. ‘Shall we do X?’) and permission seeking requests (e.g. ‘Can I do X?’). To train simulators effectively, and to illustrate to HCPs the effectiveness or otherwise of alternative strategies, several examples of each kind of practice are needed. For the CABS process, it is also important that the video clips preserve the basic sequential order of the interaction, so for example a clip needs to show both a request being made *and* how it is responded to. Clips also need to be relatively short for use in training, to ensure that trainees are able to focus on the same aspect of it. For instance, where a request for co‐operation was made three times in different ways before it was accepted, we chose to show this as three shorter clips rather than one longer one. Everyone included in the clips (or their consultee) had the opportunity to view them and consented to their use in training.

### 3.2 Facilitation

We used two facilitators to run the training, and suggest that this is the minimum amount of facilitation necessary for a CABS approach, which can then include one person with knowledge of CA, and one with experience of working with simulators. It is also desirable, as for our VOICE intervention, that both facilitators have experience of healthcare education, and of delivering care in relevant settings. Experienced facilitators are crucial in providing a safe and supportive learning environment, for example in managing (potentially delicate) feedback in such a way that it becomes a learning event for the recipient.

### 3.3 Preparing training scenarios

A key method for training actors to portray simulated patients is to give them scenarios, which provide information about the particular patient they are being asked to portray. Our CABS method also used scenarios, but with some important differences. A traditional scenario will contain a ‘backstory’ for a patient, so that a typical backstory might contain information such as ‘Mrs Jones is a 73 year old retired teacher, admitted following a fall at home. She has two children and three grandchildren and lives alone since the death of her husband. She was a keen cyclist’. We did provide this kind of backstory, based on (anonymised) real patient data. However, we also provided additional details to help with the conduct of the actual simulated interaction and to maximise its authenticity. These details included relevant information about the patient's manner of speaking, for example if s/he talked quickly or quietly, smiled a lot, or tended to echo what the HCP says. We also included suggested responses, comprising possible ways in which the patient had been observed to respond to specific kinds of questions. This information was provided alongside information about any communicative impairment that existed, and given the context of our specific study, information about the patient's retained abilities. Given the aim of maximising authenticity, it is absolutely core for the CABS method that scenarios should be based on the original video data. It is therefore important to develop each scenario based on a real person from the video data so that actors can watch and learn from these videos in preparing for the simulation. Scenarios were reviewed by a clinical expert for accuracy (e.g., in medical terminology) and plausibility before they were finalised. An example of a finalised scenario provided to actors (referred to as a ‘Level 2’ scenario because of the amount of detail it contains) is given in Table 2.

**Table 2:** Level 2 scenario for simulators from VOICE for dementia.

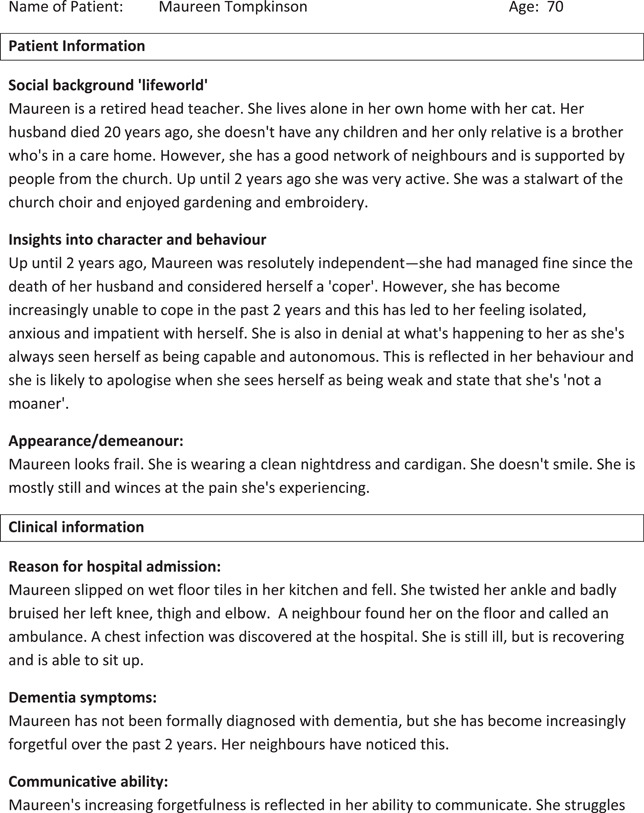		
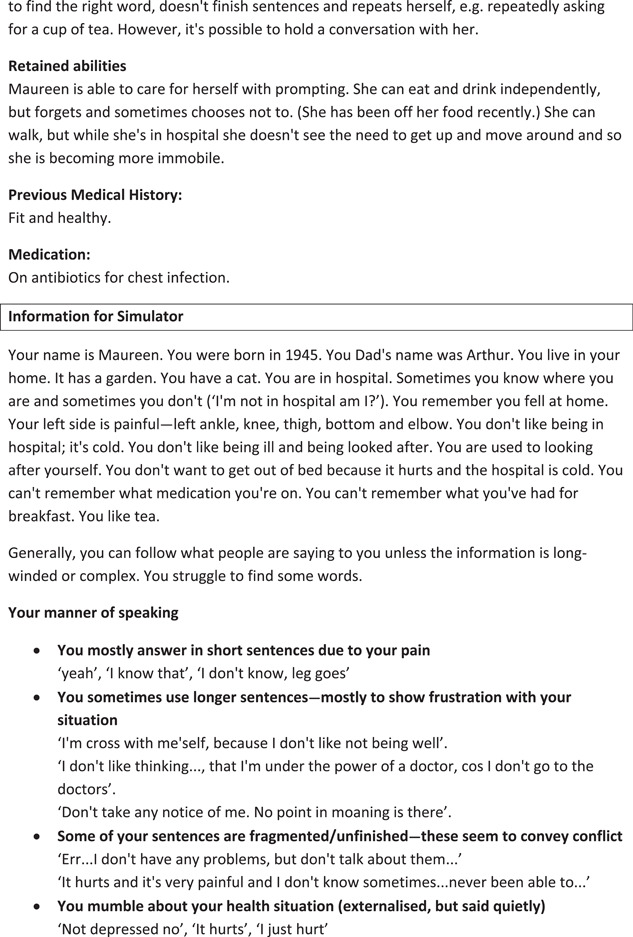		
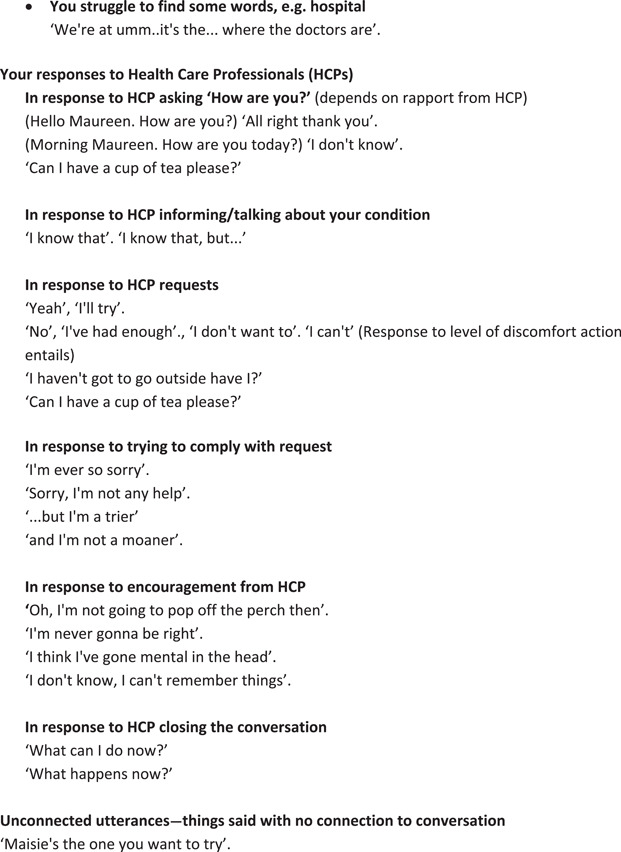		

### 3.4 Training for simulators

The actors who participated in the delivery of our intervention were chosen to fit the profile for the roles they were asked to portray, in terms of age range and sex, for face validity. All our actors were experienced in healthcare simulation, but not necessarily in this specific context. To provide them with some background information, we asked them to watch the film ‘Today is Monday’ (https://vimeo.com/93365033), made as part of a previous research project, which depicts a typical day on a ward where dementia patients are treated. We also asked them to complete some online training resources produced by the project team on the topic of dementia. A HCP with experience of working with people living with dementia was present at the training to answer any questions the actors had about symptoms, behaviour or care. Providing this background is an important component of the method and similar principles should be followed if CABS is used in other settings: for example, links to NHS websites or to support organisations could be provided to ensure a background knowledge of relevant health conditions. However, whilst these resources are useful in providing a broader background to a specific setting, and a baseline knowledge of the medical condition, a foundational principle of CABS is that actors should base their simulations around interactional practices that have occurred in real life, as captured on video recordings, rather than doing what they think a person with a particular condition might do. With the support of a senior simulator (MM) who acted as a simulator coach alongside the research team, we emphasised to the trainee simulators that they should respond to HCPs in real time, in ways that they had observed in the videos. This could include using key phrases that had been utilised by real patients, such as specific responses to requests or attempts to close an interaction. Examples of interactional practices could be drawn from more than one video; for example one might provide useful interactional detail in relation to requests, while another might provide material for closings. Since we were training HCPs to support them in dealing with situations where there could be interactional difficulty, it was important that simulators gave them practice in trying out the learning. As an illustration of this, in a scenario where a HCP was trying to get a patient to co‐operate with taking a sip of water, a simulator needed to refuse the initial request so that the HCP had an opportunity to try out some of the approaches identified as effective in the data. After two or more refusals the simulator might then decide that the HCP had had sufficient opportunity to demonstrate the trainables, and agree to the request.

Our simulator training took place over one full day. Our experience suggests that the presence of an experienced simulator/simulator trainer was critical in bridging the gap between simulators and clinicians, as they were able to observe and feed back on the simulators' performances and ensure that they did not stray too far from the scenarios, grounded in an awareness of their educational purpose. Simulators watched the video footage of all of the individual patients on which the scenarios were broadly based, alongside going through the CA transcripts with members of the research team. They were encouraged to focus on patient demeanour and mannerisms as well as what patients actually said, to underline that it was not only important to aim for authenticity in *what* was said, but also *how* it was said. Asking simulators to learn at least two scenarios ensures that HCPs have the chance to practice a range of skills. Scheduling is an important component of CABS and we allowed one week between the simulator training day and the delivery of the training course: this is important so that simulators can be allowed to process the training, develop possible responses, and reflect upon the distinct nature of the approach. Given that simulators in healthcare settings will often be asked to portray challenging situations, it is important they are supported through this time and are able to ask questions as they arise.

### 3.5 Simulation workshops for healthcare practitioners

Previous research suggests that simulation workshops are most effective in small groups, as interaction between learners is important but participating learners also need to feel safe and comfortable^27^; CABS allocates no more than 6 participants to each simulator and no more than 12 participants to each workshop. Workshop design embeds an experiential learning process, based on Kolb's learning cycle whereby trainees learn through a process of doing, reflecting, and observing.^28^ While a variety of educational theories have been used as the basis for CST interventions, it was agreed that an experiential learning approach was most appropriate for our setting, and Kolb's cycle is frequently used by clinicians in their own reflective practice. As an example, we began our workshops by showing trainees examples of requests and refusals, using our video data to demonstrate more or less successful ways of making requests, and drawing on our CA analysis to explain why some were more or less effective. Introducing HCPs to these at the outset means that they have an opportunity to practice them in the simulation that follows.

Since we trained HCPs from a variety of backgrounds, meaning there was no one task that would be a routine part of all of their care delivery, and to promote a learner‐centred approach, we allowed trainees a choice of task to carry out with the simulated patient. However, all tasks needed to be completable in a short time frame, and carried out with a very limited amount of equipment. Appropriate equipment might include a stethoscope, a glass of water, or a blanket. Examples of possible tasks from the VOICE for dementia intervention are shown in Table 3.

**Table 3:** Examples of possible tasks.

Example tasks for the VOICE for dementia intervention
Transferring a patient from a bed to a chair
Getting a patient to have a drink or something to eat
Listening to a patient's chest
Washing a patient's face (or helping them to wash it)
Asking a patient to carry out an appropriate physical activity (e.g. standing, walking).

As the aim of the CABS approach is to maximise authenticity, trainees are advised that if something is not in the room, they should not mime or pretend. For example, if there are no tea‐making facilities, a HCP should not pretend to make a cup of tea. These are important points in terms of credibility, and in terms of encouraging the HCP to behave in the simulation as they would in everyday practice.

For the simulation exercise, trainees need to be divided into small groups of roughly equal size, with one group per simulation and scenario, and one facilitator per group. The facilitator can then introduce the simulation, stressing the safe environment, the opportunity to take time out (to pause the simulation, to think or ask for advice from the rest of the group), or to repeat, and the principles for giving feedback to each other. A version of the scenario prepared specially for the HCPs can be then distributed, alongside feedback sheets for the observers, before the first volunteer chooses an appropriate task and participates in the simulation.

CABS scenarios for HCPs included only the type of information that a HCP might have access to if they were meeting a patient for the first time, for example the patient's name, reason for being in hospital, and background information such as a brief social history or details of general communicative ability. These were labelled ‘Level 1’ scenarios because they contained less detail than the scenarios provided to the actors. An example of a Level 1 scenario given to HCPs is included in Table 4.

**Table 4:** Level 1 Scenario for Trainees in VOICE for dementia.

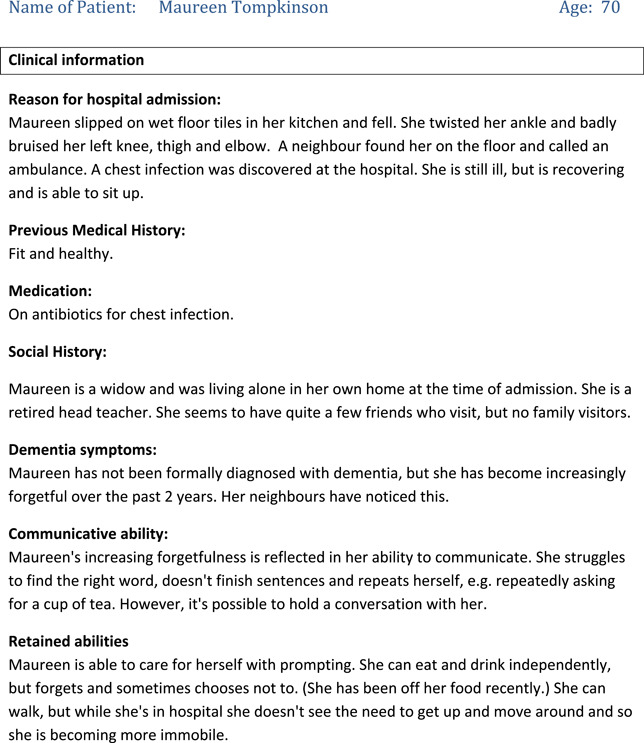		

As we have noted above, training simulations were designed to give the volunteer sufficient opportunity to practice the trainable behaviours, for example by the simulator refusing a request several times. Having other trainees observing the interaction is also an important part of the CABS method. Being able to observe and identify communication practices in others is an important part of learning, whether seeing it well done or not. This is the watching aspect of Kolb's learning cycle^28^: as Lane and Rollnick^29^ ^,p.14^ put it ‘The learner can enhance their own learning of communication skills by critically evaluating the performance of others’. Feedback sheets are also used to focus feedback on specific areas: an example of a Feedback sheet used in the VOICE study is provided in Table 5.

**Table 5:** Example of feedback sheet for simulations used in the Voice for Dementia study.

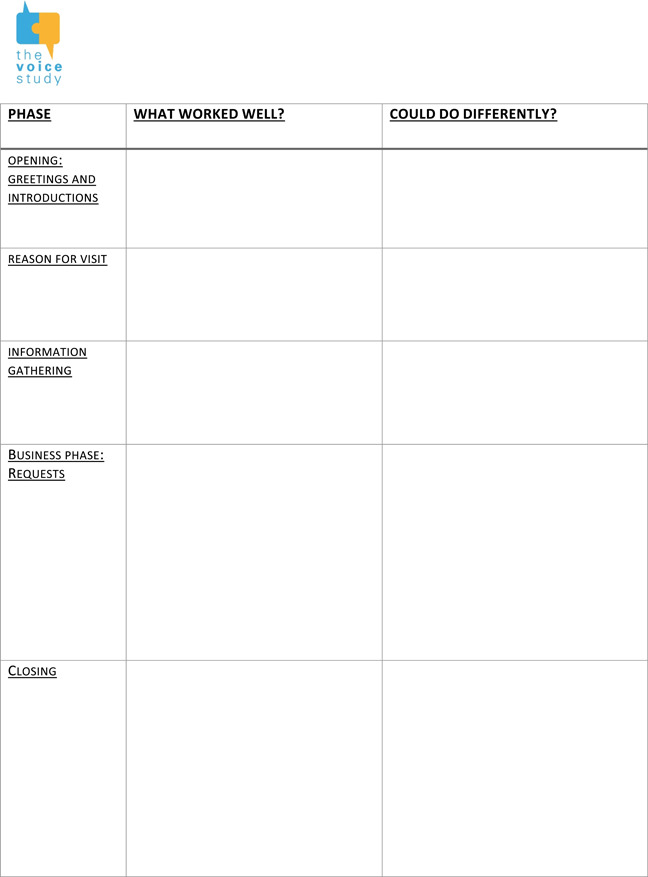		

For the simulation phase of the training, around 30 min per trainee was allowed, with the simulated interaction ending either after successful completion of the task or after 10 min had elapsed. The 30‐min timeslot enables the trainee to perform the simulation task incorporating the learned approaches, discuss how the interaction has gone, and also allows sufficient time for the feedback discussion. This time period also allows trainees to reattempt a task or part of a task if they want to, or to pause and ask for suggestions from the other trainees.

### 3.6 Feedback

Effective feedback is crucial to the learning process, and we were guided in the development of CABS by Pendleton's model of giving feedback,^30^ so that both what went well and what trainees could do better were identified. After the simulation task was over, the facilitator asked everyone to silently reflect on it for a short while. The HCP volunteer was then asked for their views on how the simulation had gone, before seeking feedback from both the observers and the simulated patient. The facilitator subsequently summarised the good practice used in the simulation alongside any ideas for improvement. By exploring specific aspects of the interaction, the learning that has taken place can be demonstrated. Highlighting what went well is important because it is well established that if someone is asked to ‘do more of something they are already doing well, you are more likely to see behaviour change and success than if you only point out what they are doing poorly’ (p. 220).^31^ It is also important because trainees may be unaware of what they are already doing that works well, and this was a point that was often returned to in course evaluations. Participants to an interaction are not generally able to describe and articulate in useful detail the practices that they have used. As Goffman^32^ ^,p.74^ argues, people often ‘act better than they know’, so their knowledge of their own interactional practices remains at a tacit level.

### 3.7 Feedback from simulators

Feedback from simulators, which is given out of role, also needs to be specific and evidence based (e.g. ‘When you said X I felt…’). It is absolutely key that the simulators are fully conversant with the trainables, and understand that their feedback should not contradict these. The simulator is also in a unique position to feed back on more general issues such as establishing rapport or empathy, for example noting whether an interaction felt rushed, or reassuring.

### 3.8 Debrief and action planning

The final component of CABS is to bring the whole group back together into the training room to discuss what has gone well during the training day, and what could be improved. This debrief allows facilitators and simulators to carry out their own reflections following Day 1 of the training, and to make any necessary changes for Day 2.

### 3.9 Training Day 2

In the VOICE for dementia intervention, we ran the training over 2 days separated by 1 month, asking participants to keep a reflective diary noting whether and how they had been able to put the trainables into practice in their roles in between the 2 days. This period of separation and reflection is important for the effectiveness of CABS. The second day reviewed reflective diaries, plus a video‐transcript workshop (which gave trainees opportunity to identify all the trained practices happening in one of the recorded encounters), and a further simulation session. As part of action planning towards the end of the training, participants were asked to identify internal/external barriers/facilitators to implementation of their newly acquired skills. The advantages of this staggered model include the opportunity to increase the difficulty of the simulated scenarios on the second day, though in some settings it may be more appropriate to focus on another trainable. In the use of CABS for the VOICE for dementia intervention, we were able to increase the level of communication impairment of the patient in the Day 2 scenarios. Participants were asked to complete a feedback form at the end of Day 2, which we discuss below.

### 3.10 Evaluation of the CABS approach

Six full training courses were delivered and formally evaluated as part of the VOICE intervention development which used CABS: 44 HCPs participated in both days of the course with a further HCP attending for 1 day only. We used a range of evaluation measures, as we have reported elsewhere; these included a questionnaire on confidence in dementia communication^33^; a dementia communication knowledge test developed by the research team; and participants' satisfaction with the training courses, including free text comments. Video‐recorded, simulated assessments were subject to masked ratings by SLTs and PPI representatives (two people with early dementia and four carers for people with dementia) to measure changes in communication behaviour. As we have reported elsewhere,^8^ these measures showed increased knowledge of dementia communication (mean improvement 1.5/10; 95% confidence interval 1.0–2.0); increased confidence in dementia communication (mean improvement 5.5/45; 95% confidence interval 4.1– 6.9; *p* < .001) and positive evaluations for the course as a whole. However, what is most relevant for the wider use of CABS as a method is that 1 month later participants reported using the skills learned in clinical practice. Masked‐ratings of simulated patient encounters confirmed these self‐reports. 2 min of each recording was selected, with the order of delivery randomised. Ratings were made independently by two trained and experienced SLT raters who were asked to rate the presence of the trained communication behaviours. SLTs are professionally trained to analyse and evaluate communication, and our raters received specific training on the relevant CA findings and trainables for the intervention.^8^ PPI raters were asked to rate the emotional tone of the encounter using a scale designed to measure person‐centredness.^34^ The results demonstrated behaviour change in taught communication behaviours to close an encounter, consistent with the training. In addition, the free text section of the course evaluation feedback form contained many comments from participants which underlined both the importance of simulation as a general tool for training, and also identified the ways in which CABS was different from more standard simulation practice. Written feedback from trainees reported views that the simulations were a safe space to try things and get them wrong and provided an opportunity to reflect on performance; this was appreciated because it was recognised it was often not possible in the clinical setting. Other responses made reference to the specific benefits of using a CA analysis to underpin the simulations, for example, by noting that the use of video recorded examples enabled participants to identify skills that they might already use but were not consciously aware of. Actors who portrayed the simulated patients reported that the novelty of the approach initially required more practice than traditional ‘backstory’ approaches, and highlighted the need for all three components of the preparatory materials (written scenario, CA transcript and video footage) to assist them; however they also reported feeling able to successfully deliver the simulations with this preparation in place.

## 4 DISCUSSION

We have outlined here the CABS method for enhancing the authenticity of simulation practice in healthcare, and illustrated this with examples of the way we applied it in a specific setting. The CABS method has some parallels with Stokoe's CARM method,^1^, ^6^ in that both are underpinned by a conversation analytic approach, and in both methods trainees are shown videos and transcripts of real interactions to help them identify which communicative approaches can work well in a particular setting. However, what is unique about CABS is that trainees are then able to put their learning into practice in simulated interactions which are closely based on data from real interactions; rather than being grounded in what patients *might* do in a particular context, they are grounded in what they *actually* do. Based on Kolb's learning cycle^28^ CABS incorporates different ways of learning: “experience (feeling); reflective observation (watching); abstract conceptualisation (thinking); and active experimentation (doing), in an ongoing process” (p. 102).^35^ Both ratings of pre‐ and post‐training interactions, and HCP feedback, suggest that the CABS method can be an effective and acceptable way of delivering CST.

## 5 CONCLUSION

In line with previous research highlighting the value of simulation as a training tool for healthcare practice,^7^ feedback from the evaluation of training sessions showed that CABS simulations provide a safe space for HCPs to try things, to be able to get them wrong in a low‐stakes environment, and to have another chance to improve them. However, there was another significant benefit reported by trainees in the course evaluations described above. The use of CABS enabled them to identify and articulate effective practices which they were already using, but had remained at a tacit level: they became aware not only that they sometimes acted ‘better than they knew’^32^ but also how. The ability of CABS to make the tacit explicit meant that they were able both to better understand why some of their existing practices were effective, and also explain these practices to others who they might have responsibility for training. Consequently, the CABS approach has the benefits of Stokoe's CARM method, in terms of using the insights that CA can provide in studying real interactions. However, it also brings the benefits of simulated interaction in terms of the ability to practice communication skills in real time, in a way that maximises authenticity.

## 6 LIMITATIONS

Some AHPs were less familiar with simulation as a standard part of professional training, and found this approach challenging at the outset. Experienced actors were required to play the role of simulated patients, and this has cost implications for delivery. This was a relatively small‐scale study in a specific area of clinical practice; however, we believe the underlying principles of the approach could be utilised across healthcare settings.

## AUTHOR CONTRIBUTIONS

All the authors made significant intellectual and practical contributions to the research project from which the process described in this article was developed. Alison Pilnick wrote the original manuscript. All authors reviewed a draft and inputted feedback or edits. Alison Pilnick and Rebecca O'Brien prepared the final submission. All authors have read and agreed to the published version of this manuscript.

## CONFLICT OF INTEREST STATEMENT

The authors declare no conflict of interest.

## Data Availability

The paper describes a process rather than an analysis. Video data from the VOICE for dementia project cannot be shared due to ethical constraints.
